# Letter-Like Shape Recognition in Preschool Children: Does Graphomotor Knowledge Contribute?

**DOI:** 10.3389/fpsyg.2021.726454

**Published:** 2022-02-16

**Authors:** Lola Seyll, Alain Content

**Affiliations:** Laboratoire Cognition Langage et Développement, Centre de Recherche Cognition et Neurosciences, Université Libre de Bruxelles (ULB), Brussels, Belgium

**Keywords:** letter representation, letter recognition, letter categorization, handwriting, graphic motor programs, visual analysis, perceptual variability

## Abstract

Based on evidence that learning new characters through handwriting leads to better recognition than learning through typing, some authors proposed that the graphic motor plans acquired through handwriting contribute to recognition. More recently two alternative explanations have been put forward. First, the advantage of handwriting could be due to the perceptual variability that it provides during learning. Second, a recent study suggests that detailed visual analysis might be the source of the advantage of handwriting over typing. Indeed, in that study, handwriting and composition –a method requiring a detailed visual analysis but no specific graphomotor activity– led to equivalent recognition accuracy, both higher than typing. The aim of the present study was to assess whether the contribution of detailed visual analysis is observed in preschool children and to test the variability hypothesis. To that purpose, three groups of preschool children learned new symbols either by handwriting, typing, or composition. After learning, children performed first a four-alternative recognition task and then a categorization task. The same pattern of results as the one observed in adults emerged in the four-alternative recognition task, confirming the importance of the detailed visual analysis in letter-like shape learning. In addition, results failed to reveal any difference across learning methods in the categorization task. The latter results provide no evidence for the variability hypothesis which would predict better categorization after handwriting than after typing or composition.

## Introduction

New technologies are pervasive in our everyday life and computers are increasingly used at school (Wollscheid et al., 2016). The possibility of typewriting replacing handwriting from the very outset of literacy acquisition thus raises the question of its impact on reading development and on written language perception. Indeed, handwriting requires to reproduce a visual form by the execution of a sequence of fine movements that completely define the shape of the letter. This activity thus incurs very precise processing in terms of both visual and motor activity. By contrast, typewriting consists in a simple keypress based on the visual matching between two graphic forms. Interestingly, recent data suggest that simple visual exposure—even if massive—is not sufficient to yield a representation sufficiently detailed to lead to successful recognition. Wong et al. (2018) examined knowledge about the shape of the “looptail” *g* allograph that is used in most print materials. They observed that skilled adult readers failed on simple tasks such as drawing the letter *g* or identifying it among distractors. Even more surprisingly, despite extensive visual exposure to the looptail *g* allograph, many skilled readers failed to simply recall its existence as an alternate form of the lowercase letter g. The authors suggested that the absence of writing experience with the looptail *g* might be the source of its ensuing underspecified shape representation. These observations question the type of experience required to construct detailed and accurate shape representations of letters, with clear educational implications.

Indeed, letter recognition ability is an important predictor of subsequent reading skills (Näslund and Schneider, 1996; Scanlon and Vellutino, 1996; O’Connor and Jenkins, 1999; Lonigan et al., 2000; Foulin, 2005). Moreover, most current models of word recognition assume that letter recognition is an essential step in the flow of processing leading to word identification (McClelland and Rumelhart, 1981; Coltheart et al., 2001; Dehaene et al., 2005; Perry et al., 2007).

Longcamp and colleagues directly assessed the impact of typewriting on the construction of letter representation. They conducted behavioral studies that compared recognition performance observed after handwriting and after typewriting (Longcamp et al., 2005b,^2006^, ^2008^). For both preschool children and adults, learning new characters through handwriting led to better recognition and orientation discrimination than learning through typewriting (see Seyll et al., 2020 for similar results). According to Longcamp et al. (2006), the advantage of handwriting is due to the contribution of the graphic motor programs—i.e., mental descriptions of the sequence of fine movements required to write the letter (van Galen, 1991; see Palmis et al., 2017, for a review)—constructed in memory through writing experience. More precisely, “the detection of a match or a mismatch between the perceived shape and the memorized motor program might contribute to the mirror–normal recognition processes and therefore explain the behavioral facilitation for the characters learned by handwriting” (Longcamp et al., 2006, p. 653). This process would be particularly helpful for letters that are ambiguous for the visual system such as mirror letters (e.g., b-d or p-q).

This interpretation thus assumes that joint reading and writing activities would gradually lead to a multimodal network of letter representation linking both the visual and the graphic motor codes (see for reviews Longcamp et al., 2010, 2016; James, 2017). In this embodied cognition perspective, one single sensory modality is sufficient to activate the entire distributed network which was engaged when the object was initially stored in memory (Wilson, 2002; Barsalou et al., 2003; Barsalou, 2008; Sullivan, 2018). The multimodal account of letter representation is supported by neuroimaging studies showing that the visual perception of letters activates precisely the same premotor area which is engaged during writing (Longcamp et al., 2003, 2005a; James and Gauthier, 2006). This premotor activation would reflect the automatic activation of the graphic motor program necessary to produce the perceived letter (Longcamp et al., 2003, 2005a).

Recently, however, several additional explanations of the advantage of handwriting over typing have been proposed. James and Engelhardt (2012; see also Li and James, 2016; James, 2017) proposed that handwriting would lead to broader and more abstract letter representations than other learning methods because it entails perceptual variability during learning. According to this hypothesis, facing varying exemplars would help identify the critical invariants and ignore irrelevant changes. The importance of perceptual variability during learning is not a new hypothesis. Indeed, according to several studies, comparison would play a critical role in the categorization of novel objects (e.g., Gentner and Namy, 1999; Namy and Gentner, 2002; Graham et al., 2010; Twomey et al., 2014). Some studies even suggested that the greater the variability among exemplars during learning, the better the generalization to new category instances (Posner and Keele, 1968; Perry et al., 2010). Handwriting is particular because in that case, the brain, the body, and the environment interact in a circular way (Li and James, 2016). The efferent motor commands sent by the brain for producing a given letter lead to variable outputs—i.e., the handwritten productions—which in turn constitute variable environmental inputs for the visual system and reshape the letter category boundaries. In contrast to handwriting, typing does not provide such variability since it exposes the learner to one single and invariant prototypical exemplar of each character, at both output and input levels.

Indeed, a recent behavioral study shows that variability improves the learning of letters by revealing that 5-year-old children were better at letter categorization when they were exposed to multiple exemplars of the letters during learning than when they were exposed to one single exemplar, whatever the learning modality—free handwriting, tracing, or viewing (Li and James, 2016). However, the advantage of perceptual variability arose whether the learning modality involved graphomotor activity or not. Moreover, it emerged whether the exemplars were self-produced, produced by another child, or simply typed. Hence, those results suggest that it is not the graphomotor activity *per se* that is the key factor explaining the facilitating effect but rather the perceptual variability that handwriting produces during learning.

A third interpretation has been proposed by Seyll et al. (2020). They suggested that the role of one component process, namely, the detailed visual analysis involved in handwriting, might have been underestimated in the advantage of handwriting over typing. More precisely, they assessed whether the detailed visual analysis required by handwriting but not by typing might contribute to the advantage of the former. To that purpose, they introduced a third learning method—called composition—requiring a detailed visual analysis but suppressing the graphomotor activity. During composition, participants received the set of elementary visual features used to construct the symbols and they were asked to reproduce the target symbol by selecting the appropriate features and assembling them together (as with a jigsaw puzzle).

After learning, participants performed two recognition tests, a four-alternative forced-choice (4AFC) test and an old/new test. Distractors used in the 4AFC were visually close to the target symbol (e.g., mirror-reversed symbol and symbol with one feature mislocated). Distractors used in the old/new recognition test consisted exclusively in the mirror-images of the learned symbols (as in Longcamp et al., 2006, 2008). The results failed to reveal any clear advantage of handwriting over composition, both leading to better recognition than typing, thus suggesting a significant contribution of the detailed visual analysis in the advantage of handwriting over typing.

We recently discovered that a similar hypothesis had already been proposed and put to the test by Courrieu and De Falco (1989).^1^ They examined perceptual discrimination of Roman letters before and after learning. The learning conditions varied according to two orthogonally manipulated factors, analysis of target letters into segments, and dynamic tracing of the letters. In the analysis condition, which is very similar to our composition condition, preschool children received the target letter broken down into three segments on separate pieces of paper, and they had to recombine the pieces to reproduce the model. In the tracing condition, the children had to draw the target letter by following a trace on the worksheet. They improved significantly more from pre- to post-test when the learning involved analysis of letters into segments than when it did not, thus supporting the detailed visual analysis hypothesis, but the results failed to reveal any clear beneficial effect of tracing. However, tracing is known to be less effective than free copying (Naka, 1998). According to Naka (1998), the disadvantage of tracing over free copying might be due to the fact that, in contrast to handwriting, tracing does not require to generate and hold the image of the shape into memory. However, it is also likely that tracing does engage the graphomotor system to a lesser extent than free copying and does not lead to the storage of a graphomotor plan robust enough to facilitate discrimination. Nevertheless, given Naka’s (1998) results, our choice of free copying provides a more appropriate test of the role of graphomotor knowledge.

The purpose of the present study was twofold. First, it aimed at further investigating the role of the detailed visual analysis inherent to handwriting by assessing whether the results observed with adults by Seyll et al. (2020) were generalizable to preschool children. To that purpose, children learned new symbols through handwriting, through typing, or through composition. After learning, they performed a 4AFC recognition task (as in Seyll et al., 2020). At the onset of reading acquisition, the visual system has to develop specific adaptations in order to effectively discriminate one letter from another. Indeed, learning to read impacts certain natural properties of the visual system. One such property is mirror-image generalization, or *mirror-invariance*, the natural process of generalization across mirror images (Bornstein et al., 1978; Biederman and Cooper, 1991; Dehaene et al., 2010). In contrast to literate adults, prereaders have not yet overcome mirror-invariance and often apply it to graphic shapes (Gibson et al., 1962; Cornell, 1985; Fernandes et al., 2016). As suggested by Longcamp et al., 2006, handwriting might be particularly helpful in overcoming mirror-invariance during literacy acquisition. If the graphic motor programs constructed through handwriting are the source of its advantage (*graphomotor hypothesis*), one might expect better recognition performance after handwriting than after typing and composition, both leading to equivalent recognition levels. In contrast, if the source of the handwriting advantage is the detailed visual analysis required by copying (*visual analysis hypothesis*), handwriting and composition should lead to equivalent recognition performance, both better than typing. Of course, the two hypotheses are not exclusive. If both graphic motor programs and detailed visual analysis contribute to the handwriting advantage (*mixed hypothesis*), handwriting should lead to more accurate recognition than composition, itself better than typing.

The absence of a clear advantage of handwriting over composition in Seyll et al. (2020) does not allow one to conclude that handwriting and composition would lead to equivalent performance in any recognition situation. Based on Li and James (2016) suggestion, it seemed plausible that despite equivalent recognition performance, handwriting would induce larger and richer representational categories as it is the only method that provides a diversity of exemplars during learning in the present experiment. It is worth noting that the 4AFC and Old/New tests may not be adequate to assess the richness of representations. The second purpose of the present study was to assess whether handwriting would improve categorization, as proposed by Li and James (2016). To this end, we also administered a categorization task like the one used by Li and James (2016). If perceptual variability improves the richness of letter representations, handwriting should affect categories and lead to better categorization performance than both other learning methods.

## Materials and Methods

### Participants

Sixty-nine French-speaking kindergarteners (5 years 3 months to 6 years 3 months) took part in the experiment. There were 35 girls and 34 boys, and four left-handed participants. All were attending kindergarten in three different schools and had no known neurological diseases or psychological disorders. Participants were randomly assigned to one of the three groups. One group learned the symbols by handwriting, the second group by typing, and the third group by composition. Data from eight participants were discarded because they did not complete all the tests. There were 20 remaining participants in the handwriting group (*mean age* = 68.1 months; *SD* = 3.68), 22 in the typing group (*mean age* = 68.5 months; *SD* = 2.92), and 19 in the composition group (*mean age* = 69.5 months; *SD* = 3.61). Written informed consent was provided by the parents. The study was approved by the local ethics committee.

### Stimuli

**FIGURE 1:**
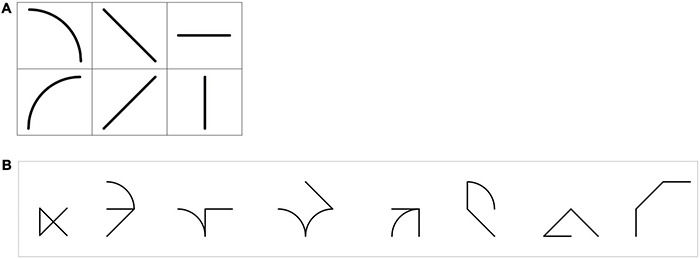
**(A)** The six elementary features used to construct the symbol library. **(B)** The eight symbols to be learned.

The method description is largely similar to Seyll et al. (2020) as the method is almost identical. Stimuli were symbols created from a set of six elementary features (Figure 1A). All possible symbols combining three features were generated, and we choose eight symbols in this library (Figure 1B). Stimuli were simpler than those used in Seyll et al. (2020). First, symbols were simplified in terms of the number of features. Indeed, whereas symbols used in Seyll et al. (2020) were composed of three, four, or five features, those used in the present study were all composed of three features. Then, only two elementary curves were used instead of four. Symbols used in the present study are referred to as “letter-like shapes” because they share the main characteristics with letters. They are the result of a combination of graphic elementary features, they can be handwritten, and the elementary features can be isolated.

### Procedure

Three sessions were held 1 week apart. The learning phase was distributed over the first two sessions. The 4AFC task was administered immediately after the second learning session and again at the beginning of the third session. The categorization task was administered during the third session immediately after the 4AFC task. Before the first learning session, participants performed visuo-spatial and graphomotor tests—i.e., the visual perception and the motor coordination subtests of the VMI, respectively (Beery and Beery, 2004). All sessions took place in a quiet room at school and participants were tested individually.

In order to promote engagement, the tasks were embedded in a treasure hunt context. A little boy was displayed on the tablet screen and children were invited to help him to find a treasure. They were then explained that this little boy is living in a very distant country and that to help him, they would have to learn the letters used in his country. Moreover, to introduce the three sessions, children were shown a three-part totem and explained that each session would be rewarded by a part of it. Once the totem was completed, they could reach the treasure.

Stimulus presentation and response recording were programmed in Python using PsychoPy libraries. Stimuli were displayed on a Wacom Cintiq 13HDT tablet.

### Learning Phase

All participants were asked to memorize eight unfamiliar symbols. In each learning session, there were three blocks each involving one random presentation of the eight symbols. Participants could take a break between blocks if needed. Before training, they received three practice trials with simple geometric shapes (a semicircle, a square, and a triangle). Feedback was given after each practice trial but not during learning.

**FIGURE 2:**
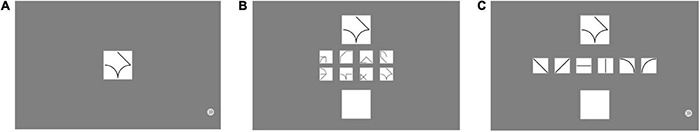
One example of trial display for each learning method: **(A)** handwriting, **(B)** typing, and **(C)** composition.

The target symbol was horizontally centered on the tablet screen (as can be seen in Figure 2, its vertical position varied as a function of the learning method) and was displayed in black in a white 37-mm-wide area against a gray background. It stayed visible on the screen during the whole trial and the transition to the next trial was triggered by the participant. No constraint was imposed on production speed.

#### Handwriting Method

The target was centrally displayed during the entire trial (Figure 2A). At the start of each trial, participants were given a 100 × 100 mm sheet, and had to copy one symbol per sheet within a square of 35 × 35 mm. Once the copy was done, the experimenter took the sheet back and hid it from view. No constraint was imposed on stroke direction or order. To trigger the next trial, participants clicked on the “next” button displayed in the lower-right corner of the screen. Response times from target onset until the “next” button press were recorded.

#### Typing Method

The screen was divided into three portions: the target symbol was displayed in the upper portion, the virtual keyboard in the middle portion, and the response area in the lower portion (Figure 2B). The virtual keyboard was composed of eight 17-mm-wide keys, corresponding to the eight target symbols. The position of the keys varied randomly across trials in order to promote an active visual research. The response area was of the same size and color as the target area. Those three portions were displayed during the entire trial. Participants had to find the key corresponding to the target symbol and click with the stylus on it. Responses triggered the apparition of the selected symbol in the response area for 1 s before the start of the next trial. Accuracy and response times from target onset until the key press were recorded. It should be noted that the typing method used in the present experiment is different from a typical typing task given that the position of the keys varied randomly across trials. In what follows, however, we will refer to it as “typing method” for the sake of clarity.

#### Composition Method

The screen was divided into three portions: the target symbol was displayed in the upper portion, the set of individual features in the middle portion, and the response area in the lower portion (Figure 2C). The middle section was composed of six features displayed in 20-mm-wide squares. The position of the features was kept constant across trials and across participants. The response area was of the same size and color as the target area. Those three portions were displayed during the entire trial. Participants had to compose the target symbol by selecting features in the features area and dragging them in the appropriate position in the response area. No constraint was imposed on stroke order. To trigger the next trial, participants clicked on the “next” button displayed in the lower-right corner of the screen. Productions and response times from target onset until “next” button press were recorded.

### Recognition Task

**FIGURE 3:**
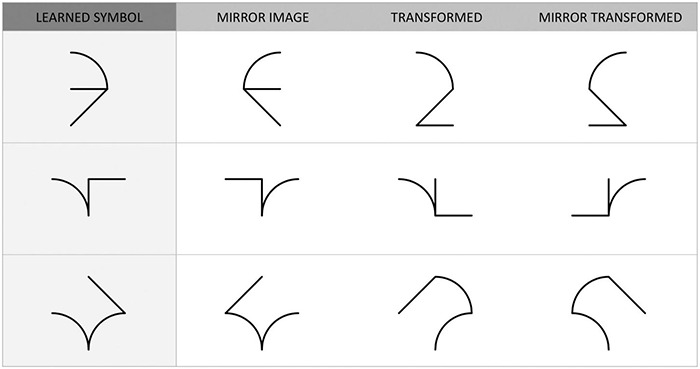
Examples of trials in the 4AFC recognition task.

Participants performed the 4AFC recognition task immediately after training (Immediate Test) and again 6–8 days later (Delayed Test). Each trial consisted of the presentation of four symbols: the learned symbol plus three distractors, i.e., the mirror image of the symbol (mirror symbol), the learned symbol with a feature displaced (transformed symbol), and the mirror image of the transformed symbol (mirror transformed symbol) (Figure 3). The four symbols were randomly displayed upper left, upper right, lower left, and lower right. The learned symbol could not occur more than twice in a row at the same position. Participants had to select the learned symbol by clicking on it with the stylus.

As for the learning phase, symbols were displayed in black in a white 37-mm-wide area against a gray background. There were two blocks each involving the random presentation of the eight symbols and their distractors. Participants could take a break between blocks.

Each trial started with a centered fixation cross for 300 ms, followed by a 200 ms gray screen. Then the four choices were displayed until the response. The intertrial interval was 500 ms. The main dependent measure was accuracy. Response speed was not emphasized, although response times from target onset were also recorded.

### Categorization Task

**FIGURE 4:**
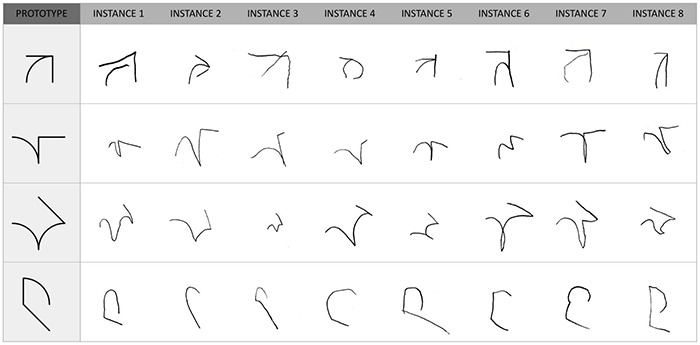
The 32 handwritten productions used as experimental stimuli in the categorization task.

During the categorization task, children were required to sort 32 handwritten exemplars into categories corresponding to four of the learned symbols. Eight exemplars of each symbol were used. They were handwritten productions created by children of the same age range in a previous study (Figure 4). The exemplars were displayed in black against a white background. Categories were instantiated by five house pictures displayed next to each other on the top of the screen. Four houses were assigned to the learned symbols, and one house was dedicated to the unlearned, new symbols. Across participants, the position of the four symbol-houses was randomized, but the “new symbols” house was fixed to the right. For a given participant, the position of the five houses remained constant. There were four blocks, each involving the random presentation of eight stimuli (two instances of each symbol). Participants could take a break between blocks.

**FIGURE 5:**
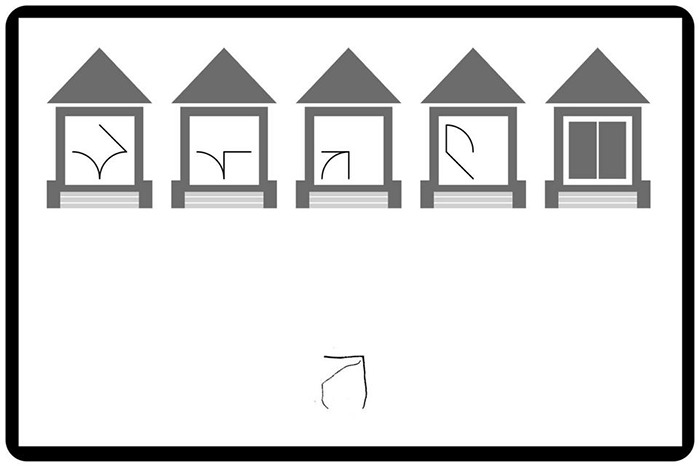
Categorization task: example of display.

Each trial started with the presentation of a new handwritten instance centered in the lower part of the screen. The five categories and the symbol instance remained visible during the entire trial (see Figure 5 for one example of trial). To select the category corresponding to the handwritten symbol, children had to click with the stylus on the corresponding house. This action triggered the instance’s move to the selected house. The trial finished by the instance’s entrance in the selected house and a short blast. If the child did not identify the symbol as belonging to any of the four symbol categories, he could select the “new symbols” category. The main dependent measure was accuracy. Response speed was not emphasized, although response times were also recorded.

Before the test, participants performed 10 practice trials with four simple geometric shapes categories (a circle, a heart, a square, and a triangle). Oral feedback was given after each practice trial but not during the test.

## Results

All data files are available at https://osf.io/a2893/. In frequentist analyses, handwriting was systematically contrasted to typing on the one hand and to composition on the other hand (as in Seyll et al., 2020). For both tasks, accuracy was analyzed in terms of proportion of correct responses. As no emphasis was put on response times, they were not further analyzed. Response times on correct trials were around 5,100 ms for both tasks, and they were similar for all learning methods.

### 4AFC Recognition Task

**FIGURE 6:**
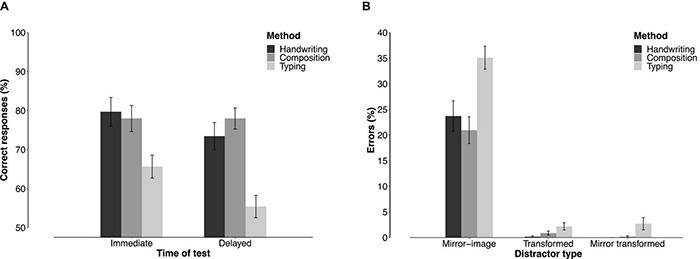
**(A)** Mean percentage of correct responses for the immediate and delayed 4AFC test across learning methods. **(B)** Errors produced across the three learning methods. Error bars depict standard errors.

Shapiro–Wilk normality tests on percentages of correct responses indicated no significant deviation from normality. Correct response rates (see Table 1) were submitted to an ANOVA with learning method (handwriting, composition, typing) as a between-subject factor and time of test as a within-subject factor (immediate, delayed). Mean percentages of correct responses are plotted in Figure 6A. The main effect of learning method was significant, *F*(2,58) = 15.006, *p* < 0.001, η*_*p*_*^2^ = 0.341. *A priori* contrasts revealed a significantly higher proportion of correct responses after handwriting (*M* = 76.1%, *SD* = 13.5) than after typing (*M* = 60.0%, *SD* = 9.03), *t* = 4.525, *p* < 0.001, but no significant difference between proportion of correct responses following handwriting and composition (*M* = 78.0%, *SD* = 11.5), *t* = 0.380, *p* = 0.705. The main effect of time of test was significant, *F*(1,58) = 6.200, *p* = 0.016, η*_*p*_*^2^ = 0.097, reflecting a higher rate of correct responses immediately after learning (*M* = 74.1%, *SD* = 16.0) than 1 week later (*M* = 68.3%, *SD* = 16.8). The interaction was not significant, *F*(2,58) = 1.816, *p* = 0.17, η*_*p*_*^2^ = 0.059. To assess evidence in favor of an absence of difference between the handwriting and composition conditions, we additionally ran a Bayesian repeated-measures ANOVA, which produced concordant indications. Overall, the best model included time of test and learning method (BF_10_ ∼ 10,000). *Post hoc* comparisons provided decisive evidence of differences between typing and both other conditions (Kass and Raftery, 1995, respectively, BF_10_ ∼ 50,000 for composition and BF_10_ ∼ 2,500 for handwriting) and substantial evidence in favor of an absence of difference between composition and handwriting (BF_10_ ∼ 0.254).

**TABLE 1:** Performance for both recognition tasks across learning methods.

		Composition	Handwriting	Typing
**4AFC task**				
**Immediate test**				
	Mean percent correct responses	78.0%	79.7%	65.6%
	Standard deviation	14.6%	16.3%	13.9%
**Delayed test**				
	Mean percent correct responses	78.0%	73.4%	55.4%
	Standard deviation	11.9%	15.6%	13.4%
**Mirror errors**				
	Mean percent mirror errors	20.9%	23.7%	35.1%
	Standard deviation	11.4%	13.3%	10.4%
**Categorization task**				
	Mean percent correct responses	71.1%	73.3%	68.2%
	Standard deviation	15.8%	14.4%	18.4%
	Mean percent “New” errors	24.0%	22.5%	26.0%
	Standard deviation	18.6%	15.3%	21.9%

Error types are plotted in Figure 6B. On average, participants selected the mirror-image of the learned symbol on 26.9% of trials, the transformed symbol on 1.1% of trials, and the mirror transformed symbol on 1.0% of trials. A Shapiro–Wilk test on the rate of mirror-image choices indicated no significant deviation from normality. An ANOVA performed on the rate of mirror-image choices revealed a significant difference between learning methods, *F*(2,58) = 8.561, *p* < 0.001, η*_*p*_*^2^ = 0.228. A significantly higher proportion of mirror-symbol choices was observed after typing (*M* = 35.1%, *SD* = 10.4) than after handwriting (*M* = 23.7%, *SD* = 13.3), *t* = 3.144, *p* < 0.001, but there was no significant difference between handwriting and composition (*M* = 20.9%, *SD* = 11.4), *t* = 0.734, *p* = 0.466. The Bayesian ANOVA provided strong evidence in favor of an effect of learning method, BF_10_ ∼ 58. *Post hoc* tests again indicated differences between typing and both other conditions (BF_10_ ∼ 11 and 136, respectively, for handwriting and composition), and weak evidence in favor of the absence of difference between composition and handwriting (BF_10_ ∼0.377).

### Categorization Task

Shapiro–Wilk normality tests on percentages of correct responses indicated significant deviations from normality in two of the three groups. Hence, scores were submitted to a non-parametric ANOVA with learning method (handwriting, composition, and typing) as a between-subject factor. The Kruskal–Wallis test was non-significant [*H*(2) = 0.863, *p* = 0.65]. A Bayesian ANOVA similarly provided substantial evidence in favor of the null (BF_10_ ∼0.195). Mean percentages of correct responses and error rates are reported in Table 1.

On average, participants selected an erroneous category on 5.0% of trials and the “new symbols” category on 24.2% of trials. Shapiro–Wilk normality tests on rates “new symbols” choices indicated significant deviations from normality in two of the three groups. A non-parametric ANOVA performed on the rate of “new symbols” choices revealed no significant difference between learning methods, *H*(2) = 0.226, *p* = 0.89. The Bayesian ANOVA produced substantial evidence in favor of the null (BF_10_ ∼0.15).

## Discussion

The changing habits introduced by the increasing use of digital devices in everyday life and at school raises the question of their impact on literacy acquisition. Indeed, one might wonder whether the reduced usage of handwriting at the very outset of reading acquisition has an impact on letter recognition, an essential step in word identification (McClelland and Rumelhart, 1981; Coltheart et al., 2001; Dehaene et al., 2005; Perry et al., 2007) generally considered as predictive of subsequent reading skills (Näslund and Schneider, 1996; Scanlon and Vellutino, 1996; O’Connor and Jenkins, 1999; Lonigan et al., 2000; Foulin, 2005).

A negative impact of keyboard use during letter learning has already been demonstrated. Indeed, multiple behavioral studies showed that handwriting is a more effective learning method and leads to better recognition and mirror discrimination than typing (Longcamp et al., 2005b,^2006^, ^2008^; Seyll et al., 2020). Several interpretations of this finding have been proposed. First, the motor knowledge acquired through handwriting could contribute to recognition. Second, the perceptual variability entailed by handwriting could be the source of its advantage (James, 2017). Third, Seyll et al. (2020) argued that the detailed visual analysis required by handwriting might be a significant factor accounting for the advantage of handwriting over typing.

The first aim of the present study was to assess whether the results observed with adults by Seyll et al. (2020) were generalizable to preschool children. To that purpose, children learned new graphic shapes through handwriting, through typing, or through composition, and performed a 4AFC recognition task after learning. The second aim was to assess whether handwriting would lead to better categorization than typing and composition. To that purpose, we added a categorization task like the one used by Li and James (2016) at the end of the last session.

Recognition performance in the 4AFC task confirmed and corroborated the results observed in adults (Seyll et al., 2020), that is, higher recognition rates after handwriting and composition than after typing, with the two former leading to equivalent performance. Such a pattern is consistent with the idea that the detailed visual analysis plays an important role in letter-like shape learning and provides no evidence that the graphic motor programs, as such, contribute to letter recognition. Moreover, the present findings are in line with most current models of word recognition, which assume that letter recognition is a visual process based on elementary features extraction (McClelland and Rumelhart, 1981; Coltheart et al., 2001; Perry et al., 2007; Grainger et al., 2008). Indeed, the composition learning method used in the present study can be linked to the latter models because it precisely involves a visual focus on elementary features during learning.

Regarding the second issue, correct categorization rates failed to reveal any significant difference across the three learning methods. Moreover, if handwriting leads to richer and larger categories, new test exemplars should be less frequently rejected and a lower proportion of “new symbols” choices should be observed after handwriting than after typing and composition. However, as for the main analyses, the percentage of “new symbols” choices failed to reveal any significant difference across learning methods. Our results thus provide no evidence to confirm the variability hypothesis.

Mirror discrimination is essential for efficient reading. The present findings replicate the detrimental impact of typing on mirror-normal discrimination observed in previous studies (Longcamp et al., 2005b,^2006^, ^2008^; Seyll et al., 2020). Mirror-image errors are common at the onset of reading acquisition and dramatically decrease in the course of reading acquisition, between 5 and 7 years of age (Gibson et al., 1962; Nelson and Peoples, 1975; Fernandes et al., 2016). Several studies suggest that reversal errors are more frequent in children with developmental dyslexia during the first years of schooling (Wechsler and Hagin, 1964; Liberman et al., 1971; Wolff and Melngailis, 1996), and a recent study suggest that dyslexic children do not automatize mirror discrimination (Fernandes and Leite, 2017). There is thus cause for concern about a possible exacerbation of this weakness with the introduction of keyboarding at school. Dyslexic children might be more impacted by learning through typing than typically developing children and the predominant use of typing at school might constitute an additional risk factor for them.

In sum, the present findings clearly confirm that the detailed visual analysis is important in letter-like shape learning. It would yield detailed, accurate, and distinctive representations which support easy discrimination and identification. Under such a view, the association between letter perception and motor activation should be interpreted as a consequence of the learning experience and not as a necessary condition for encoding and recognition. Neither the present nor our previous studies (Seyll et al., 2020, 2021) showed an advantage for handwriting over composition and the Bayesian inference tests supported the null hypothesis. Even if it is too early to completely discard a possible contribution of graphic motor programs to letter recognition, our findings challenge the supporters of the graphomotor hypothesis to provide further evidence, over and above the influence of detailed analysis.

Regarding the potential implications of our conclusions for educational issues, it should be noted that the present learning situation differs in several ways from the usual school settings: the learning task was strictly visual and did not involve associations between graphic shapes and letter names or sounds, and the artificial symbols used here differ from real letters. Further studies using more ecological conditions and stimuli would be relevant to confirm the present findings.

While the present results provide no evidence in favor of a contribution of graphic motor programs and handwriting *per se*, it should, however, be noted that in the classroom, handwriting training and copy exercises may constitute the most natural way to promote such detailed visual analysis for most kids. Yet, handwriting might not constitute a suitable learning method for children with poor fine motor skills. Indeed, several studies revealed that poor fine motor skills are associated with poor reading skills (e.g., Grissmer et al., 2010; Cameron et al., 2012; Suggate et al., 2018, 2019). With normal adults, Seyll and Content (2020) showed that disturbing the graphomotor activity during symbol learning affects subsequent recognition and mirror-normal discrimination. The advantage of composition over typing observed in the present study might be exploited with children suffering from severe fine motor skills deficits. Indeed, it is plausible that children with poor fine motor skills would benefit from composition learning, as did the children of the present study. Further studies would be necessary to determine whether this is indeed the case.

## Data Availability Statement

All data files are available at https://osf.io/a2893/.

## Ethics Statement

The studies involving human participants were reviewed and approved by Comité d’Éthique de la Faculté des Sciences Psychologiques et de l’Éducation, ULB. Written informed consent to participate in this study was provided by the participants’ legal guardian/next of kin.

## Author Contributions

Both authors designed the experiment, analyzed the data and wrote the manuscript.

## Conflict of Interest

The authors declare that the research was conducted in the absence of any commercial or financial relationships that could be construed as a potential conflict of interest.

## Publisher’s Note

All claims expressed in this article are solely those of the authors and do not necessarily represent those of their affiliated organizations, or those of the publisher, the editors and the reviewers. Any product that may be evaluated in this article, or claim that may be made by its manufacturer, is not guaranteed or endorsed by the publisher.

## Footnotes

1 We thank the Editor for pointing us to that reference.
